# *De novo* mutational profile in *RB1* clarified using a mutation rate modeling algorithm

**DOI:** 10.1186/s12864-017-3522-z

**Published:** 2017-02-14

**Authors:** Varun Aggarwala, Arupa Ganguly, Benjamin F. Voight

**Affiliations:** 1 Genomics and Computational Biology Program, Perelman School of Medicine, University of Pennsylvania, Philadelphia, PA 19104 USA; 2 Department of Genetics, Perelman School of Medicine, University of Pennsylvania, Philadelphia, PA 19104 USA; 3 Department of Systems Pharmacology and Translational Therapeutics, Perelman School of Medicine, University of Pennsylvania, Philadelphia, PA 19104 USA; 4 Institute for Translational Medicine and Therapeutics, Perelman School of Medicine, University of Pennsylvania, Philadelphia, PA 19104 USA; 5 Perelman School of Medicine, University of Pennsylvania, 415 Anatomy Chemistry Building, 3620 Hamilton Walk, Philadelphia, PA 19104 USA; 6 Perelman School of Medicine, 10–126 Smilow Center for Translational Research, University of Pennsylvania, 3400 Civic Center Boulevard, Philadelphia, PA 19104 USA

**Keywords:** Mutation Rate, Retinoblastoma, *de novo* mutations, Variability in Mutation Rate, Variant Prioritization

## Abstract

**Background:**

Studies of *de novo* mutations offer great promise to improve our understanding of human disease. After a causal gene has been identified, it is natural to hypothesize that disease relevant mutations accumulate within a sub-sequence of the gene – for example, an exon, a protein domain, or at CpG sites. These assessments are typically qualitative, because we lack methodology to assess the statistical significance of sub-gene mutational burden ultimately to infer disease-relevant biology.

**Methods:**

To address this issue, we present a generalized algorithm to grade the significance of *de novo* mutational burden within a gene ascertained from affected probands, based on our model for mutation rate informed by local sequence context.

**Results:**

We applied our approach to 268 newly identified *de novo* germline mutations by re-sequencing the coding exons and flanking intronic regions of *RB1* in 642 sporadic, bilateral probands affected with retinoblastoma (RB). We confirm enrichment of loss-of-function mutations, but demonstrate that previously noted ‘hotspots’ of nonsense mutations in *RB1* are compatible with the elevated mutation rates expected at CpG sites, refuting a RB specific pathogenic mechanism. Our approach demonstrates an enrichment of splice-site donor mutations of exon 6 and 12 but depletion at exon 5, indicative of previously unappreciated heterogeneity in penetrance within this class of substitution. We demonstrate the enrichment of missense mutations to the pocket domain of *RB1*, which contains the known Arg661Trp low-penetrance mutation.

**Conclusion:**

Our approach is generalizable to any phenotype, and affirms the importance of statistical interpretation of de novo mutations found in human genomes.

**Electronic supplementary material:**

The online version of this article (doi:10.1186/s12864-017-3522-z) contains supplementary material, which is available to authorized users.

## Background

Studies of *de novo* mutation offer new potential to elucidate the etiology of both Mendelian and complex human diseases [1], made increasingly possible by efficient, large-scale re-sequencing of the coding portion of the human genome. This class of mutations can lead to the identification of disease-causal genes [2–5] and etiological pathways [6, 7], help to refine the underlying genetic mechanism and architecture [8], and ultimately can aid in clinical management of disease for mutational carriers.

After a causal gene has been identified, it is natural to hypothesize that disease relevant mutations accumulate within a sub-sequence of the gene – for example, an exon, a protein domain [9], or at CpG sites [10]. Previous studies of *de novo* mutational burden for complex disease have largely focused on gene or pathway discovery, and have benefited from statistical models that capture base-pair variability in the mutation rate [6, 11, 12]. However, because hundreds of genes are implicated for an individual complex disease, and owing to sizes of these studies which typically number in the hundreds to a few thousands subjects [8], the number of *de novo* events per gene is small and thus limits the power to infer pathogenicity of sub-sequences within the gene. In contrast, for Mendelian diseases that are not extremely rare and where the genetic architecture is less complex (*i.e.,* one or a few genes are disease causal), *de novo* mutational burden concentrates to individual genes [13], facilitating the possibility of genic sub-sequence characterization. However, previous efforts have largely been enumerative rather than quantitative, as improved models of mutation for the human genome [14] and a large-scale collection of genetic variation segregating in the coding genomes of human populations have only been recently described [15].

Progress in investigating hypotheses of mutational burden within sub-sequences has been hampered by the lack of accurate models that capture mutation rate variability in human genomes at base-pair resolution. Previous studies have utilized approaches based on enrichment of *de novo* mutations in disease ascertained samples to infer pathogenicity [16–18]. However, because sub-genic sequences can introduce germline mutations more frequently due to a higher intrinsic rate of mutation, it is critical to model variation in mutation rate to accurately detect enrichment at sub-sequences [19]. Recently, we described a statistical model for nucleotide substitution using local sequence context, which explains a substantial fraction of variability in mutation rates observed in human populations [14]. In what follows below, we describe an approach that facilitates direct hypothesis testing for an enrichment of *de novo* mutations within the sub-sequence of a gene, beyond that expected from our mutational model at base-pair resolution. Our report here differs from important, recent work demonstrating the functional intolerance to new mutations found in the protein domains of genes [9], with application targeted toward variant prioritization for locus discovery in human disease. In addition, our approach differs from existing tools like TADA or Poisson models [12, 20], which are designed to assess the total mutational burden in a gene. In contrast, our approach directly tests for the enrichment of *de novo* mutations in disease ascertained samples over part of gene suspected to harbor pathogenicity (*e.g.*, protein domains, exons, specific amino acids, etc.) against a null hypothesis reflecting the background variable rate of mutation across a gene. Our objective is to assess if the distribution of mutations *already observed* is itself unusual, heterogeneous in space across a gene or within a mutational class. As a proof of concept, we apply our testing framework on a data set consisting of *de novo* mutations discovered in 642 newly re-sequenced patients affected with sporadic, bilateral Retinoblastoma (RB). RB is an extensively studied cancer of the developing retina, and the distinctive clinical features of bilateral tumors and a younger age at diagnosis is associated with the presence of germline mutations in the tumor suppressor retinoblastoma 1 (*RB1*) gene [21].

In RB, it is not fully understood if *de novo* mutations occur uniformly over *RB1*, or instead localize to specific codons, sequence contexts, or protein domains. Based on Knudson’s model [22], we expect a higher frequency of *de novo* mutations that result in putative loss-of function (LoF) in *RB1* in patients ascertained for RB, which has been previously shown [16]. Numerous studies have reported a preponderance of nonsense mutations at CpG sites in *RB1* [10, 16, 23, 24]. These observations could suggest a role of CpG sites in generating nonsense mutations via the deamination of hyper-methylated CpGs as a potential mechanism [17, 25, 26], though this postulation remains to be statistically evaluated. In addition, numerous splice-site mutations have also been observed in *RB1* [23, 24, 27], many of which have been shown to result in exon skipping [27]. However, it remains to be quantified if mutations in all essential splice sites are equivalently pathogenic. Finally, recurrent point mutations have been observed at specific codons, which includes Arg661Trp [28–30]. This codon falls within the pocket domain in *RB1* [31], an important domain that facilitates binding of the protein product with downstream targets to regulate cell cycle. However, to our knowledge, enrichment of mutations at this or other codons in *RB1* has not been *statistically* quantified. In what follows, we demonstrate (i) that the previously reported excess of nonsense mutations in *RB1* at CpGs is compatible with the elevated rate of mutation at those sites, refuting a specific pathogenic mechanism in RB, (ii) an enrichment of essential splice-site donor mutations at exon 6 and 12, but depletion at exon 5, indicative of previously unappreciated heterogeneity in relative penetrance across this type of putative LoF mutation, and (iii) a statistically significant excess of mutations found at Arg661Trp in bilateral RB, as a hotspot for missense mutations with lower penetrance. Our approach is generalizable across disease endpoints, providing a statistical framework to characterize rare diseases with today’s data, but also expanded, complex disease studies collected in the future.

## Results

### An algorithm to quantify the enrichment of *de novo* mutations

Our central objective is to determine if the frequency, type, and location of *de novo* mutations for a given gene are consistent with the number of events predicted from our local, nucleotide sequence context model for mutation rate variability. For example, we expect more nonsense mutations in RB patients than our background model predicts, because (i) we ascertained individuals with RB, (ii) nonsense mutations are likely LoF, and (iii) LoF at *RB1* causes RB. To achieve this objective, we require an accurate model that captures variability in the frequency of *de novo* mutational events across a gene and an engine to distribute mutations in that gene according to this model. With these in place, we can empirically assess significance of enrichment of *de novo* mutations in exons or sub-sequences of *RB1* relative to our model prediction.

**Fig. 1:**
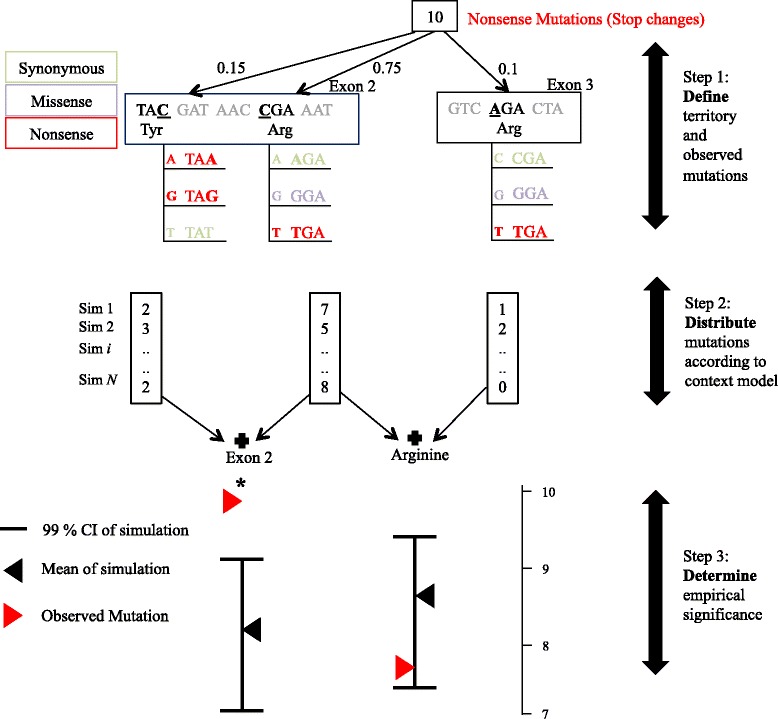
Approach to quantify if patterns of *de novo* within a mutational class are unusual. Our approach involves three steps. First, we identify the genomic target (base pair territory) in which mutations will be characterized, and the total number of mutations found in that territory. We then distribute this total number of mutations over the target territory using a background model of mutation rate. Second, we find the expected number of mutations in different categories (Exon, mutational type like Nonsense or specific Amino Acid) using the previous distribution samples. Third and finally, we compare this to the observed number of mutation to detect statistical enrichment in a category beyond expectation. In this toy example depicted here, we focus on the genomic territory that can generate nonsense mutation (shown in red), and imagine that we have identified 10 *de novo* mutations that are nonsense. First, we identify eligible base pairs and that can result in a nonsense change. Next, we calculate the probability of mutation at each eligible base pair as the sum of substitution probabilities of that sequence context changing to a stop codon (shown in red). Second, we then distribute the mutations over multiple simulations from a multinomial distribution, and find the distribution of the expected number of mutations at each of these eligible base pairs. We are particularly interested in cases where the observed number of mutations at a subclass (exon or an amino acid) is greater than what we see in simulations, as this is compatible with disease-relevant pathogenicity for this class of mutation, or position where the mutation(s) is located. Third and finally, for a particular subclass we combine the expected mutations at different eligible base pairs and compare the overall expected distribution with observed, and conclude enrichment

In our previous work [14] we demonstrated that an expanded sequence context model which considers three flanking nucleotides on either side of a base (*i.e.*, heptanucleotide), explains variation in germline mutation rate better than competing models of sequence context, and up to 93% of the variability in substitution probabilities. Using the sequence context based substitution probabilities, we developed an algorithm to distribute mutations across the gene in order to generate an expected count of mutations (with variance) at all positions in *RB1* (Fig. 1, Methods). With these distributions in hand, we can estimate the empirical significance conditioned on the observed number of any type of substitution in any sub-sequence(s) within the gene. As an imperfect control, we use singletons from ExAC (allele frequency of ~1/66,000, ~0.00152%) in which to compare our *de novo* events, with the assumption that these events are the youngest and have not experienced the full force of purifying selection; *i.e.*, are the closest proxy to *de novo* events segregating in (non-Finnish) European populations. In what follows, we apply our approach to study (i) the overall frequency of nonsense, essential splice-site, and missense mutations in *RB1* and ExAC, and (ii) their spatial occurrence by exon or by sub-sequence (CpG sites, domains, or codons).

### Re-sequencing of sporadic bilateral RB patients identifies 268 *de novo* single base point mutations

To quantify the role of *de novo* mutations in the pathophysiology in RB, we re-sequenced *RB1* in 642 cases presenting sporadic (*i.e.*, without family history), bilateral RB and their parents. Our targeted resequencing included all exons of *RB1* as well as 50 base pairs of intronic sequences on either side of exons (Methods). For statistical modeling purposes, we focused on single base point mutations and excluded individuals who carry a frame-shift or in-frame insertion-deletion mutations. After variant calling followed by quality control, we identified 276 *de novo* germline, single base point mutations (Methods). Owing to an alternative start codon in exon 1 [10, 32], our subsequent analyses focus on the remaining exons, resulting in 177 amino-acid altering mutations, 86 in essential splice-sites, and 5 mutations found in introns outside of essential splice-sites (total of 268 *de novo* events, Additional file 1: Table S1, Methods). Consistent with the causal role of *RB1*, the discovery of 268 *de novo* mutations in 642 RB probands is highly unusual (Expected number of variants = 0.1, *P* < < 10^−10^, Methods). Furthermore, we observed more nonsense and essential splice-site mutations than missense or intronic mutations, expected given the pathogenic nature of loss-of-function (LoF) mutations in *RB1* (Table 1). For a population-level comparison, we contrasted our mutational profile to the data obtained from the Exome Aggregation Consortium (ExAC) [15], consisting 60,706 individuals re-sequenced for the exome. We note that ExAC excluded childhood diseases from their aggregation, which may have excluded RB patients. As a result, we do not expect this sample to represent a completely random population sampling of mutations in *RB1*. From ExAC, we focused on singletons observed in non-Finnish populations of European ancestry (*n* = 149 variants in >33,000 subjects, Additional file 1: Table S2, Methods). Consistent with samples from ExAC as population-level controls with potential ascertainment against RB disease, we observed fewer loss-of function and more missense and intronic variants compared to our *de novo* mutations identified in RB probands (Table 1).

**Table 1:** Counts of *de novo* mutations in *RB1* ascertained from RB patients, and singleton variants identified in ExAC from (non-Finnish) Europeans for various subtypes

Variant Type	RB *de novo* mutations	ExAC singletons
Overall	268	149
Nonsense	150	1
Missense	27	56
Essential Splice	86	1
Intronic	5	91

### Abundance of nonsense mutation at CpG sites is explained by elevated mutation rate

**Fig. 2:**
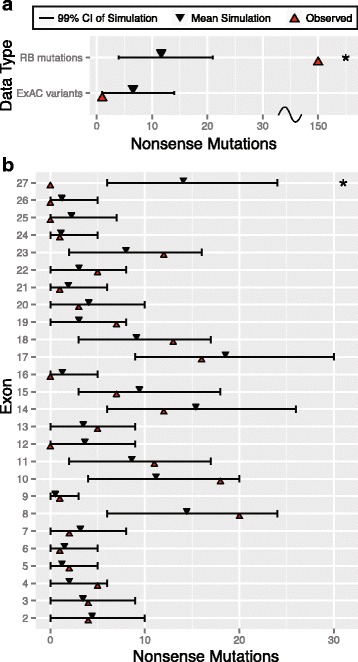
Overall and exon specific pathogenicity in nonsense mutations. **a** Comparison of the overall observed number of mutations to the simulated frequency of nonsense mutations in both RB and ExAC datasets. **b** Comparison of the observed number of mutations to the simulated frequency of nonsense mutations in RB, across exons 2 to 27. The asterisk (*) denotes that the observed number falls outside the 99% confidence interval (*i.e.*, *P* < 0.01). CI: Confidence Interval

We first investigated if nonsense mutations were distributed proportionally to the predicted rate of mutation, or alternatively localize to specific sequences, like CpGs. As a positive control, we first distributed the 268 identified mutations ascertained in RB probands and determined how many nonsense mutations we predicted from our sequence context mutational model. We found an enrichment of nonsense mutations beyond that expected from our model (*P* < < 10^−6^, Fig. 2a, Methods). This observation is consistent with extensive literature showing that LoF mutations at *RB1* cause RB. As a negative control, we distributed variants identified from the ExAC database, and observed fewer nonsense mutations than expected based on our model (*P* = 0.0103, Fig. 2a, Methods). This is also expected, as we anticipate few (if any) nonsense mutations in *RB1* observed in the general population or in ExAC that may have excluded RB patients.

We next examined if the subset of 150 nonsense mutations we observed were unusually distributed across exons in *RB1* (Methods). We found that, across virtually all exons, nonsense mutations occurred as frequently as our model predicts, broadly consistent with the concept that nonsense mutations found across *RB1* are similarly pathogenic (Fig. 2b). The single exception was exon 27, which segregated fewer mutations than our model predicted (*P* < < 10^−6^, Fig. 2b). This observation is compatible with the hypothesis that nonsense mutations in exon 27 are not fully penetrant, perhaps due to incomplete nonsense mediated decay [33] or that this exon may not be integral to the etiology of RB. Previous studies have observed fewer mutations at later exons in the *RB1* gene [16], though they were unable to quantify the reduction and assess statistical significance as we are able to here. While we observed fewer mutations at exons 25 and 26, these numbers are still compatible with our background mutational model, given the number of mutations that were discovered in re-sequencing.

Next, we examined if the subset of 150 nonsense mutations we observed were unusually distributed in amino acid type or codon contexts across *RB1* (Methods). We found that the distribution of *de novo* events by amino acid and codon context was not especially different from what our mutational model predicted (Table 2). Specifically, our model predicted a large number of C-to-T transitions resulting in Arginine to Stop mutations at the CGA codons (93 observed, 99% CI: 73–104, *P* = 0.24), presumably due to the higher mutational frequency at the CpG context [19, 34]. This analysis indicates that the observed profile of nonsense mutations can be explained by the background rate of mutation without a need to invoke a RB-specific mutation-promoting or pathogenic mechanism at CpG sites.

**Table 2:** Comparison of the observed number of nonsense *de novo* mutations to the simulated frequency predicted by our sequence context model

Amino Acid	99% CI of simulation	Observed variants	Empirical P
Lysine	[0, 11]	3	0.336
Serine	[2, 15]	6	0.404
Leucine	[1, 13]	5	0.454
Glutamine	[5, 23]	15	0.385
Tryptophan	[1, 13]	3	0.126
Arginine	[73, 104]	95	0.188
Glutamic	[4, 20]	14	0.243
Glycine	[0, 6]	3	0.211
Cysteine	[0, 7]	1	0.399
Tyrosine	[2, 16]	5	0.143
Arginine Codon	99% CI of simulation	Observed variants	Empirical P
CGA	[73, 104]	93	0.237
AGA	[0, 4]	2	0.209

Data shown for all amino acids which can change to a stop codon as well as Arginine codon partitioned by CpG context. *CI* confidence Interval

To replicate these observations, we repeated our analysis on an independent set of 100 nonsense *de novo* germline mutations in *RB1* identified in bilateral RB patients (Additional file 1: Table S3, Methods). These results recapitulated the observed deficiency of nonsense events in exon 27, and our model also matched the number of nonsense mutations at CpG sites or at CGA codons relative to other nonsense sites (Additional file 1: Table S4, S5).

### Excess splice-site donor mutations in introns 6 and 12, but depleted in intron 5 of *RB1*

**Fig. 3:**
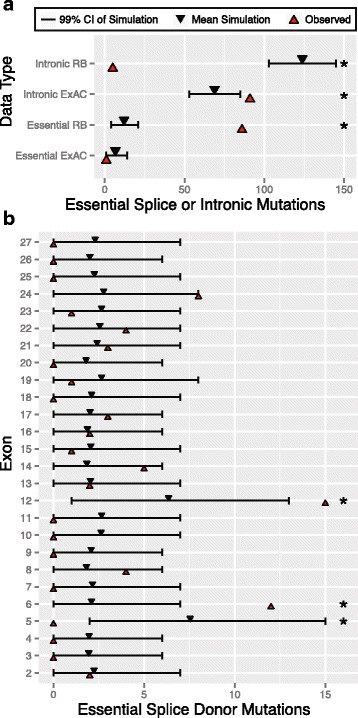
Overall and exon specific enrichment in essential splice-site mutations. **a** Comparison of the overall observed number of mutations to the simulated frequency of essential splice and intronic mutations in both RB and ExAC datasets. **b** Comparison of the observed number of mutations to the simulated frequency of essential splice donor mutations in RB, across exons 2 to 27. The asterisk (*) denotes that the observed number falls outside the 99% confidence interval (*i.e.*, *P* < 0.01). CI: Confidence Interval

We next investigated if essential splice-site and intronic mutations were distributed proportionally to the rate of substitution predicted by our context model. As a positive control, we distributed the 268 mutations ascertained in RB probands and determined how many essential splice-site and intronic mutations we expected from our sequence context mutational model. We found more *de novo* essential splice sites mutations in RB patients than predicted (*P* < < 10^−6^, Fig. 3a, Methods). This observation is consistent with the idea that essential splice-site mutations that are LoF at *RB1* cause RB. As a negative control, we distributed variants identified from the ExAC database and observed fewer essential splice variants there (*P* = 0.014, Fig. 3a, Methods). This is not unexpected: analogous to nonsense mutations described above, we anticipate few essential splice-site mutations in the general population and/or ascertainment against RB patients in ExAC participants. In intronic sequences that are found outside of essential splice sites, we observed substantially fewer events in RB patients that our model predicted (*P* < < 10^−6^, Fig. 3a). In contrast, we found more intronic events in ExAC that our model would predict (*P* < < 10^−6^, Fig. 3a). Taken collectively, these two observations indicate that intronic and essential splice-site sequences do not have a homogeneous rate of mutational ascertainment, and given that intronic mutations are ascertained less frequently, indicate lower overall pathogenicity for intronic mutations outside of essential splice-sites (Fig. 3a), as expected given that essential splice sites are generally intolerant to mutation.

We then examined if the 86 essential splice-site mutations we ascertained in RB probands were unusually distributed across introns in *RB1* (Methods). First, we found that essential splice-site acceptor mutations were not unusually distributed (Additional file 2: Figure S1), so we focused on the remaining 63 essential splice-site donor mutations. Next, we observed no mutations in the donor site of intron 5, which was outside our model prediction (*P* < < 10^−6^, Fig. 3b). However, this observation is readily explainable: if we assume that essential splice-site donor mutations here result in exon skipping as seen for other splice-site mutations [27], it turns out that skipping exon 5 retains the coding reading frame albeit with a 13 amino acid deletion (Additional file 3: Figure S2). Therefore, this type of mutation may not result in full LoF of the *RB1* protein product, and thus, may be weakly penetrant, if at all. Next, we found that essential donor splice-site mutations in intron 6 and 12 segregated more mutations that our model predicted (*P* < < 10^−6^, Fig. 3b). Previous studies have observed that exon 6 and 12 mutations are recurrently mutated in *RB1* [23, 24], though they were unable to quantify the enrichment and assess statistical significance as we are able to here.

It is not immediately apparent why these *specific* splice-site mutations are enriched in RB ascertained patients compared to other splice donor mutations. Essential donor splice-site mutations at intron 6 and 12 result in exon skipping [27], out-of frame shift mutation, and putative LoF (Additional file 3: Figure S2). However, essential donor splice-site mutations at other introns (except intron 5) also result in frame-shift mutations in *RB1* if exons are skipped. To further validate the observation of specific enrichment at these exons, we utilized the Leiden Open Variation (LOVD) Database [35] (Methods), a curated catalog of mutations found in *RB1*. Because variants are reported from multiple studies, where the gene territory re-sequenced and total number of individuals ascertained is not completely documented, we are limited in our ability to statistically quantify variant enrichment in LOVD as we can for our data. We found recurrent mutations with multiple reported variants (or fewer for exon 5) even in the LOVD [35] database of all reported variants in *RB1* gene of patients with RB (Table 3). Moreover, the donor sequences of inton 6 and 12 also are similar to other canonical splice sequences found at other (not enriched) exons. Taken collectively, these data suggest some additional pathogenic burden of these mutations relative to other essential splice-sites in *RB1*.

**Table 3:** Comparison of the observed number of essential donor splice-site *de novo* mutations at exons 6, 12, and 5 to the simulated frequency predicted by our sequence context model

Location	99% CI of simulation	Observed variants	Empirical P	LOVD count
Exon 6 (G → C)	[0, 2]	3	3 × 10^−4^	40
Exon 6 (G → A)	[0, 4]	9	<10^−6^	
Exon 12	[0, 10]	13	4 × 10^−4^	67
Exon 5	[1, 12]	0	3 × 10^−3^	2

“LOVD count” denotes the point variants observed at this site in the LOVD dataset. In Exon 6, we list separately the simulated frequency for each mutational class type (G to C and G to A). *CI* confidence Interval

### Localized enrichment of missense mutations to Arg661Trp in *RB1*

**Fig. 4:**
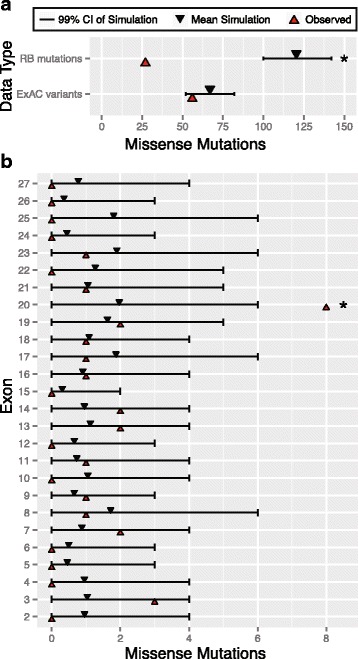
Exon specific and localized enrichment of missense mutations in *RB1*. **a** Comparison of the overall observed number of mutations to the simulated frequency of missense mutations in both RB and ExAC datasets. **b** Comparison of the observed number of mutations to the simulated frequency of missense mutations in RB, across exons 2 to 27

We investigated if missense mutations were distributed proportionally to the rate of substitution predicted by our context model. We distributed the observed 268 mutations across the gene, and found significantly fewer missense mutations than expected (*P* < < 10^−6^, Fig. 4a, Methods). This observation is consistent with the model that missense mutations as a class *generally* are less penetrant for RB, contrasting against the substantially higher penetrance of LoF nonsense or essential splice mutations. In contrast, ExAC participants were not unusual in the distribution of missense variants observed relative to our model prediction (*P* = 0.041, Fig. 4a). Taken collectively, these data suggest that, as a class, missense mutation in *RB1* are less frequently pathogenic than nonsense variants and result in fewer mutations ascertained in RB probands.

The idea that missense mutations *generally* are less penetrant for *RB1* still leaves open the possibility of heterogeneity in pathogenicity among sub-sequences of *RB1*. For example, Arg661Trp is a frequently observed mutation found in families that segregate lower penetrance [28–30]. Computational prediction tools like Polyphen2 [36] or evolutionary conservation based metrics [37] are frequently used to rank missense variants categories of deleteriousness as a proxy for pathogenicity. We applied Polyphen2 to classify all missense mutations we identified, and found most of them to be damaging (Additional file 1: Table S6).

**Fig. 5:**
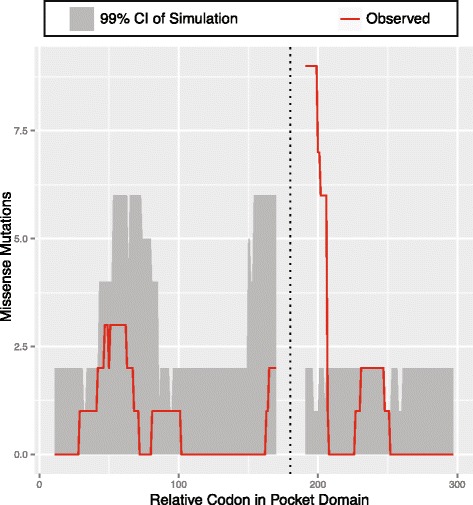
Comparison of the observed number of mutation to the simulated frequency of missense mutations over codons in the pocket domain of *RB1*. Here, a sliding window of 10 amino acids on either side of the codon was considered. Dotted line denotes the gap in the pocket domain

To further improve the resolution of these predictions, we applied our approach to identify a smaller, statistically credible subset of missense mutations implicated in RB pathogenicity. To achieve this, we distributed all 27 missense mutations we ascertained in RB probands across *RB1* to determine if these rates were proportional to our predicted mutational model (Methods). We observed a significant enrichment of missense mutations in exon 20, mapping to the known pocket domain in *RB1* (Fig. 4b, 8 mutations out of 27, *P* < < 10^−6^). Although the pocket domain in *RB1* gene encompasses other exons [29, 31] (*i.e.*, Pocket Domain Box A: Exons 13–17, Pocket Domain Box B: Exons 18–22), we did not observe a specific enrichment of missense mutations there (all *P* > 0.01, Fig. 4b). We next distributed the missense mutations within the pocket domain territory in *RB1* (*n* = 18 missense mutations in 307 codons across the entire pocket domain). We observed an excess of missense mutation burden within exon 20 in Pocket Domain Box B near codon 661 than predicted by our model (*P* < < 10^−6^, Fig. 5).

We next sought to localize the signal of the missense mutational burden within exon 20. We distributed all missense mutations we observed within exon 20 (*n* = 8 in total), and observed an enrichment of missense mutations from CGG to TGG coding for a change from Arginine to Tryptophan (Additional file 1: Table S7). Specifically, we found the previously observed recurrent mutation Arg661Trp (*n* = 5 times in our sample) occurred more frequently that our model predicted (*P* < < 10^−6^). We note the limited resolution of Polyphen2, as it also predicts other sites nearby as damaging (Additional file 1: Table S6).

To place this observation in context of other missense mutations documented in *RB1*, we evaluated the frequency of *n* = 130 missense mutations in exon 2 to 27, curated by the LOVD repository. There, the most frequently cataloged missense mutation was Arg661Trp (*n* = 33 of 127), with the next most frequently listed as C712R (*n* = 8 of 127), G137D (*n* = 6 of 127), and T307I (*n* = 5 of 13). However, when reflected against ExAC, Arg661Trp was observed only once (<0.001%) and C712R was not observed at all, consistent with putative pathogenicity of both variants. In contrast, G137D and T307I were far more frequent in ExAC (0.04% and 0.3%, respectively), suggestive of very low RB penetrance for these events. While the LOVD ascertainment is certainly complex and precludes us from formally evaluating statistical significance, these data are consistent with the importance of Arg661Trp as pathogenic and a frequently mutated position.

### Quantification of relative rates of different classes of mutations found in *RB1*

**Fig. 6:**
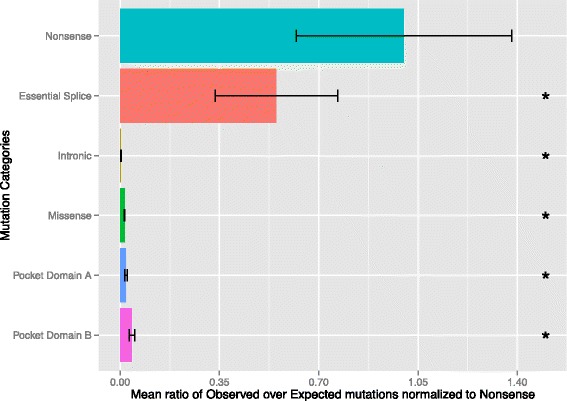
Comparison of the relative rates of different types of *de novo* mutations, normalized to the rate of nonsense mutations. Plotted is the mean of the ratio of observed number of mutations over expected based on the computational model. Mutational categories that have a different rate from the nonsense category (*P* < 0.01) are denoted by an asterisk (*). CI: Confidence Interval

Finally, we sought to quantify – relative to nonsense mutations – the rates of various sub-types of *de* novo mutations we observed in *RB1*. Assuming the penetrance of nonsense mutation is nearly full, the idea here is that if a subtype of *de* novo mutation were as penetrant as nonsense mutations, we would expect to have ascertained that subtype as frequently as nonsense mutations, proportional to the mutability of the subtype. We found that the rate of ascertainment of essential splice-site mutations was statistically lower than nonsense mutations (*P* < < 10^−10^, Fig. 6, Methods), consistent with the lower penetrance of essential splice mutations due to some less pathogenic changes observed at the essential splice positions (*e.g.*, intron 5). Similarly, the rate of intronic and missense mutations relative to nonsense was substantially smaller (*P* < < 10^−10^, Fig. 6). Finally, while the rates of missense mutations found in both Pocket Domain Box A and B were less frequent relative to nonsense mutations, we noted that mutations localized to Box B were more frequent compared to missense mutations overall or in Box A (both *P* < < 10^−10^, Fig. 6). Together, these data suggest a mixture of penetrant missense mutations found across *RB1*, elevated in penetrance for Box A mutations, and further elevated in Box B, the Box that also contains codon 661.

## Discussion and conclusions

A major challenge in *de novo* mutational studies of rare and complex disease is to not only identify new pathogenic mutations, but also to statistically quantitate the enrichment of specific types of pathogenic mutations within a gene, in order to improve the understanding of gene-specific disease etiology. To address this question, we developed a generalized approach, based on local nucleotide sequence context, to model variability in mutational probabilities at base pair resolution. Our motivation was based on the need to statistically evaluate specific hypothesis about the relative abundance – and inference about pathogenicity – of *de novo* mutations identified in probands selected for bilateral RB without a previous family history of disease. Our approach provides a strategy to statistically interpret the enrichment of specific types and location where mutations occur in genes, important as the clinical community obtains large numbers of mutations from re-sequencing and may be tempted to speculate on apparent excesses in mutational frequency without comparing to what might be expected by chance. While the mutational model utilized here is the best performing from those that are currently available [12], we expect that these models will continue to improve over time. Our proposed approach is flexible and can accommodate future, improved models. The interpretation of our findings were also clarified by contrasting our results against singleton variants identified in the largest aggregation of publicly available sequenced exomes from ExAC. One caveat here is that we assumed that observed singleton mutations were close (but imperfect) proxies to the *de novo* mutation rate. That study did observe fewer singletons than expected, suggesting the signature of recurrent mutation. Thus, while our estimates here may report fewer that the total number expected, we note that the size of *RB1*, the magnitude of the recurrent mutational imprint, and simulations suggest only a small impact on our interpretation of ExAC variation.

Our collection is both of qualitative and clinical importance. First, this study of sporadic RB cases identified under a research protocol represents the single largest dataset of *de novo* mutations in the *RB1* gene reported to date. Thus, it removes many uncertainties associated with other data sets where there are many sources of non-homogeneity including sample ascertainment and methods used for mutation detection. Moreover, the significance of identifying *de novo* mutations for affected probands includes not only clinical management decisions, but also risk of a second cancer in the future as well as having additional, affected offspring. Thus, investigating the pathogenicity of *de novo* mutations by this study is both mechanistically and clinically relevant. In terms of clinical importance, our results imply that (i) splice site mutations at exon five are likely not pathogenic, (ii) that exon 6 and 12 splice junction mutations are unusually pathogenic, and (iii) missense mutations around the pocket domain are more pathologically significant. The latter two cases may motivate further clinical monitoring or phenotypic follow-up studies to quantify future cancer risk for those specific mutations.

The analysis we present on these data helps to bring clarity to several outstanding questions in the field. First, we show that the frequency of nonsense mutations at CpG sites is compatible with our background model for the known, elevated rate of mutation at these sites. A parsimonious interpretation of this result is simply that nonsense mutations at CpG sites in *RB1* are, in fact, *not* preferentially RB pathogenic. Instead, the abundance of Arginine to Stop mutations can simply be explained by (i) ascertainment of RB affected probands, (ii) that LoF at *RB1* causes RB, and (iii) the mutability of this sequence context [14, 34]. Second, we identified heterogeneity in the frequency of essential donor splice-site mutations across *RB1*. In particular, we found a depletion of essential donor splice site in intron 5, explainable by the fact that exon 5 skipping retains the coding frame (at the cost of a 13 amino acid deletion) and thus may only be weakly penetrant. We also found more essential donor splice-sites of introns 6 and 12 than predicted by our model, which result in frame-shift and putative LoF. We note that essential donor splice-sites in other introns also result in frame-shift and putative LoF. Thus, a mechanistic explanation as to why exon 6 and 12 skipping and consequent frame-shift LoF would be *specifically* ascertained in our probands remains elusive. Nonetheless, statistical quantification of this specific enrichment, to our knowledge, has not been previously reported.

Finally, we quantified the excess of missense mutations in Exon 20, localized specifically to Arg661Trp. While we noted the recurrence of five mutations to this specific codon, as well as and enrichment in another LOVD dataset, we were not able to distinguish the relative frequency of this mutation from the rate of nonsense owing to the small number of events we ascertained. Previous reports in the literature gives some indication that this mutation is indeed low penetrance [28–30], and our results are consistent with these reports. With sufficient data and a specific, probabilistic model, it is conceivable to utilize our approach to derive posterior distributions for penetrance for this and other classes of mutations we observed. Such may be the focus of future work.

We focused here exclusively on the analysis of RB, owing to the systematic extent that this disease has been previously studied, the preponderance of existing data sets, and minimal genetic heterogeneity for the condition. Despite this, our efforts helped to clarify existing hypotheses in the field around mutational mechanisms for the gene and point to new areas to study for this already well-studied disease. That said, our framework could be readily applied for interpreting the large collection of *de novo* events in additional monogenic or oligogenic (*i.e.*, Mendelian) diseases. Or alternative, in the near future for complex disorders where genes have been identified and re-sequenced in a large number of patient populations and numerous *de novo* events have been catalogued. While each disease endpoint will have particular biological mechanisms to elucidate, the model and approach we present should provide a statistical framework to identify sequence-based features that point to unknown mechanisms underlying human disease.

### Data access

#### Patient samples

Patients included in this study were recruited as part of a research protocol between 1998 and 2011 from pediatric oncology clinics within North America. The *de novo* mutations presented here were identified from 642 children in the Genetic Diagnostic Laboratory at the University of Pennsylvania. These samples represent bilateral RB cases without family history, and where both parental DNA sample was available. Parental DNA samples were tested for the mutations identified in the respective affected child to rule out familial cases, and to unambiguously establish the presence of *de novo* mutational events. Of the 75 sporadic bilateral cases identified previously [38], only 23 samples overlap (*i.e.*, had parental samples also submitted/available).

#### DNA isolation and sequencing

The isolation of DNA, PCR amplification of *RB1* sequences, and Sanger sequencing of amplified PCR products was performed as previously described [38]. Primer sequences used for amplification are available on request.

#### *RB1* genic sequence region

We considered the genic sequence of *RB1* with accession number L11910 in the GENBANK database. Only exons 2 to 27 in *RB1* were analyzed; exon 1 was excluded to match the design of a previous study, owing to cryptic start site in the gene [32], though exon 1 mutations did not appear unusually distributed (data not shown). We also analyzed 50 base pairs on both 5′ and 3′ ends of the exon. Six base pairs on the 3′ end of the exon were defined as donor essential splice sites, while 2 base pairs on the 5′ end were defined as acceptor essential splice sites. The remaining nucleotides, from position 7 to 50 on the 3′ end of the exon, and from 3 to 50 on the 5′ end of the exon were defined as intronic sites. As a result, we analyzed a total of 5,460 nucleotide bases in the gene, out of which 2,787 were from protein coding region, 2,457 intronic and remaining 216 belonging to essential splice sites. We provide the entire annotated genomic territory (Additional file 1: Table S8).

#### RB mutational data

A total of 571 mutations were identified, which included 289 point mutations. Furthermore, we considered missense, nonsense, essential splice (six base pairs on the 3′ end of the exon were defined as donor essential splice sites, while 2 base pairs on the 5′ end were defined as acceptor essential splice sites), and intronic mutations that falling in the *RB1* sequence region defined per the above, that passed quality control. We note that the apparent difference between the total number of individuals sequenced (*n* = 642 probands) and the number of mutations found in RB1 (*n* = 571) is not dramatically different than previous reports [16, 34], perhaps suggesting additional pathogenic non-coding variation missed by our survey focused primarily on coding sequences. As a result, 268 mutations falling in our region of interest were analyzed.

#### ExAC variants

We only considered the singleton variants (missense, nonsense, essential splice and intronic) in the non-Finnish European populations from the ExAC dataset. We initially downloaded all variants in “ENSG00000139687” gene id from ExAC, including only mutations that were observed once (singletons). As a result, we analyzed 149 singleton variants falling in our region of interest as described above.

#### RB mutational data from collaborators

We independently received nonsense mutational data from a recent publication of germline *de novo* mutations in RB [10]. We analyzed 100 variants from this dataset that were present in our region of interest as described above.

#### LOVD variants

We queried the variants present in the 2015 release of the Leiden Open Variation Database (http://rb1-lsdb.d-lohmann.de/home.php?select_db=RB1) for in the *RB1* gene. We only reported the results from the point mutations present in the database.

## Methods

### Analysis of the total number of mutations discovered

Unlike the noncoding region, previous studies [39] have reported a higher *de novo* mutation rate of ~1.5 × 10^−8^ mutations per base pair per generation in the coding region. Since we consider a total genomic territory of 5,460 nucleotide base pairs in RB1 gene and sequenced 642 individuals (or 1,284 haploid chromosomes), we expect a total of 0.1 *de novo* mutations in our sample. This is calculated by multiplying the *de novo* mutation rate (1.5 × 10^−8^) with the total genomic territory (5460 base pairs) and total number of haploid chromosomes sequenced (642 × 2). Since, we observed 268 non-silent *de novo* mutations and we expect 0.1 *de novo* mutations, we report extreme statistical significance after simulations from a Poisson distribution with fixed parameter as the expected mean of 0.1.

### Mutation enrichment analysis conditional on a set of observed mutations

The majority of analyses presented in the paper focused on generating the expected number (and variance in) mutation number, conditioned on a specific type of event or sub-sequence with *RB1* where a set of events had occurred. In the case of mutations identified in RB probands, this involved distributing all (or a subset of) *n* = 268 *de novo* mutations we discovered by re-sequencing *RB1*. For the comparison to ExAC, this involves distributing all (or a subset of) *n* = 149 singleton variants we identified in non-Finnish Europeans, as an admittedly imperfect proxy for *de novo* events. Our procedure involved three steps:

- *Step One: Select genomic territory and observed mutations that fall in region of interest.* For results where *n* = 268 mutations were distributed, we considered all of the available genomic territory that was re-sequenced and filtered from our discovery effort (*i.e.*, a total of 5,460 bases, as described above). Here, if the base pair position did not result in a desired type of mutation, that base is excluded. The number (and type) of mutations that are subsequently distributed was based on those actually discovered within the specified territory. Finally, we assumed *de novo* mutations located in any/all positions in our territory was always able to be discovered, if present.
- *Step Two: Distribute mutations on sequence according to context model.* The probability of mutation at each base pair of the genomic territory selected in Step One is provided by our 7-mer sequence context based substitution probabilities, which were estimated from the non-coding genome in our prior work [14]. Briefly, a nucleotide base can change into one of three other bases (*e.g.*, nucleotide C can change to A, G, or T) with different substitution probabilities based on the type of change. Depending on the codon and position context, this nucleotide change can result in one of many types of coding changes (*e.g.*, nonsense, splice-site, etc.). The type of mutations selected in Step One determines which of these three nucleotide base changes at the position is considered. For example, if only nonsense mutations were selected in Step One, we would consider only the base pair positions and subset of possible nucleotide changes in each base pair that result in a nonsense mutation. Once all probabilities across base pairs have been identified, we then normalize by the sum of all probabilities so that the total at all eligible bases where a change could occur in the gene is 1. For a given simulation and the total number of mutations selected in Step One, each is distributed across the gene from a multinomial distribution with probabilities as estimated before.
- *Step Three: Determine Empirical Significance*. For each comparison, we performed 1,000,000 simulations to determine the empirical distribution of mutation count found at the type or sub-sequences of mutations specified in Step Two. Empirical p-values for significant enrichment (deficiency) were determined by counting the number of times that the simulations had a value greater (less) than or equal to the observed number of mutations in that class.

### Rates of different classes of mutation, relative to nonsense mutations

We first calculate the ratio of observed to expected mutations in a category after distributing all 268 *de novo* mutations at all eligible bases and possible changes (any change except those resulting in a synonymous mutation) using our algorithm described before. Next, we normalized this ratio by dividing it with the mean for nonsense category. This results in setting the mean of observed to expected variants for nonsense category as 1. We then plot the mean and standard error of this ratio for each category of mutations. The different distributions of this observed to expected rate are compared using a standard 2 sample t-test.

## Additional files

Additional file 1: Table S1. All *de novo* germline variants in *RB1* gene of patients with RB. “gDNA position” is the nucleotide position in the GENBANK accession number L11910 of the gene. **Table S2.** All ExAC variants in *RB1* gene that were considered in our analysis. “gDNA position” is the nucleotide position in the GENBANK accession number L11910 of the gene. **Table S3.** All Nonsense variants in *RB1* gene from Onadim and Houdayer groups. “gDNA position” is the nucleotide position in the GENBANK accession number L11910 of the gene. **Table S4.** Comparison of observed mutations and the simulated frequency of nonsense changes per exon, to find differential pathogenicity within nonsense mutations. Analysis was performed on data from Onadim and Houdayer groups. **Table S5.** Comparison of observed mutations and the simulated frequency of nonsense changes to find differential pathogenicity within nonsense mutations. Data shown for all amino acids and two arginine codons (99% CI) which can change to a stop codon. Analysis was performed on data from Onadim and Houdayer groups. **Table S6.** Polyphen predictions on the *de novo* germline missense mutations or some potential variants near codon 661 in *RB1* gene. “Polyphen2_format” is the variant format accepted by the Polyphen2 tool. “Polyphen_prediction” is the result of Polyphen2 on the missense variant. **Table S7.** Comparison between observed mutations and the simulated frequency of missense changes at amino acids and codons in exon 20, to find localized pathogenicity within missense mutations. Only the significant results are reported here. **Table S8.** Genomic territory of *RB1* gene analyzed in our study. “Position Start” is the start position of the entry as per GENBANK database. “Position End” is the end positon of the entry as per GENBANK database. “Annotation” is the description of the entry. Possible keywords are exon or donor/acceptor region in essential splice or nonessential intronic region. “Exon” corresponds to exon number of the entry. (XLSX 30 kb)
Additional file 2: Figure S1. Comparison of observed mutations and the simulated frequency of essential splice acceptor mutations in RB (99% CI) to find exon specific differential pathogenicity within essential splice mutations. Exons where the observed mutations are higher or lower than the 99% confidence interval of simulations are denoted by an asterisk (*). (PDF 143 kb)
Additional file 3: Figure S2. Donor splice mutations in Exons 5, 6 and 12, and their effect on codon structure. The codon structures are shown prior and after the donor splice mutation. The donor splice mutation results in exon skipping or deletion, but can also cause a frameshift mutation in certain cases. (PDF 84 kb)
