# Supplementary figures and images for: De novo mutational profile in RB1 clarified using a mutation rate modeling algorithm
Source: BMC Genomics. 2017 Feb 14;18:155. doi: 10.1186/s12864-017-3522-z (PMC5307739; doi:10.1186/s12864-017-3522-z)

Additional file 2: Figure S1

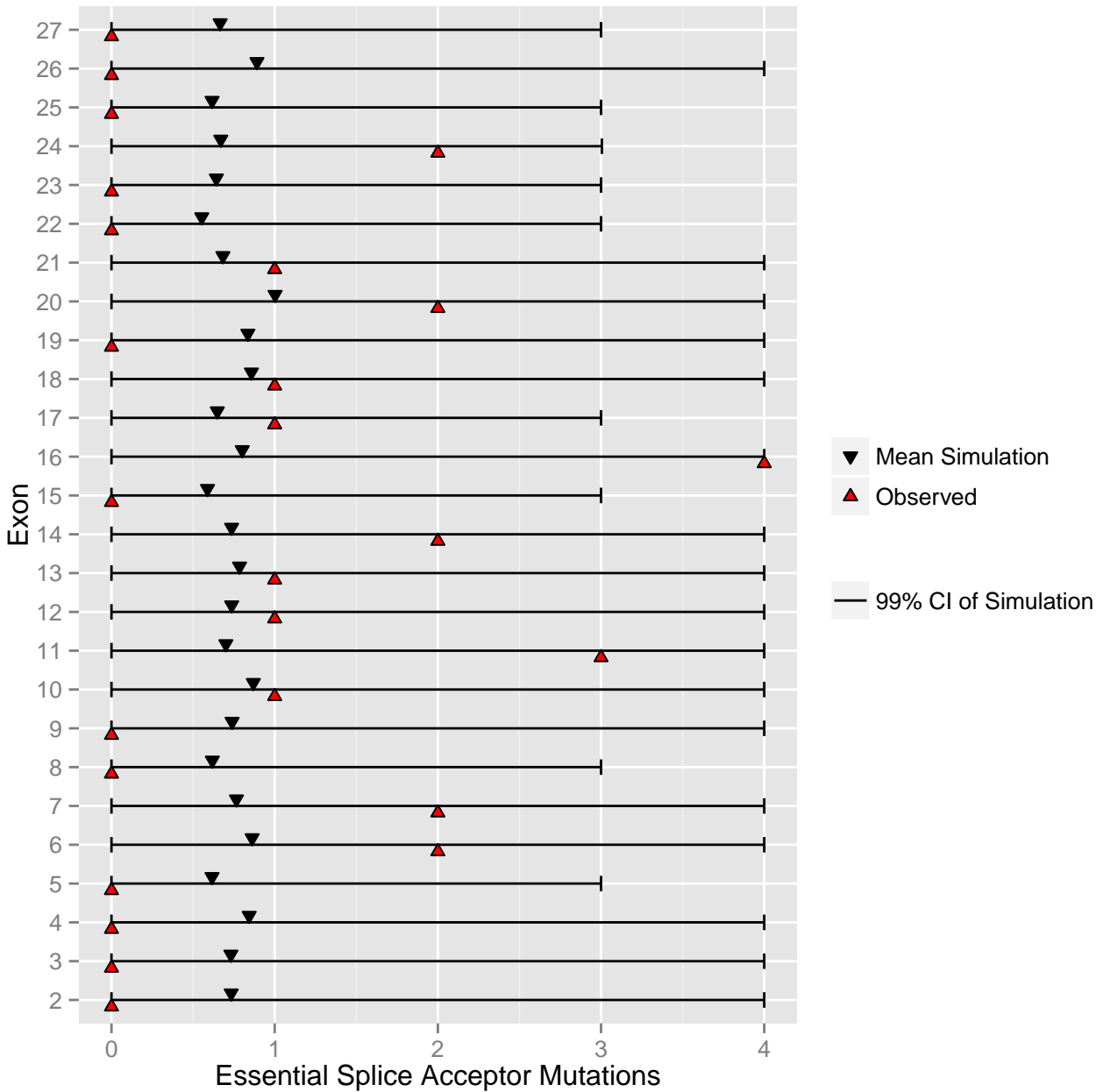

Supplement: Additional file 2: Figure S1. — Comparison of observed mutations and the simulated frequency of essential splice acceptor mutations in RB (99% CI) to find exon specific differential pathogenicity within essential splice mutations. Exons where the observed mutations are higher or lower than the 99% confidence interval of simulations are denoted by an asterisk (*). (PDF 143 kb) [file 12864_2017_3522_MOESM2_ESM.pdf]
