# Supplementary material for: De novo mutational profile in RB1 clarified using a mutation rate modeling algorithm
Source: BMC Genomics. 2017 Feb 14;18:155. doi: 10.1186/s12864-017-3522-z (PMC5307739; doi:10.1186/s12864-017-3522-z)
Supplement: Additional file 3: Figure S2. — Donor splice mutations in Exons 5, 6 and 12, and their effect on codon structure. The codon structures are shown prior and after the donor splice mutation. The donor splice mutation results in exon skipping or deletion, but can also cause a frameshift mutation in certain cases. (PDF 84 kb) [file 12864_2017_3522_MOESM3_ESM.pdf]

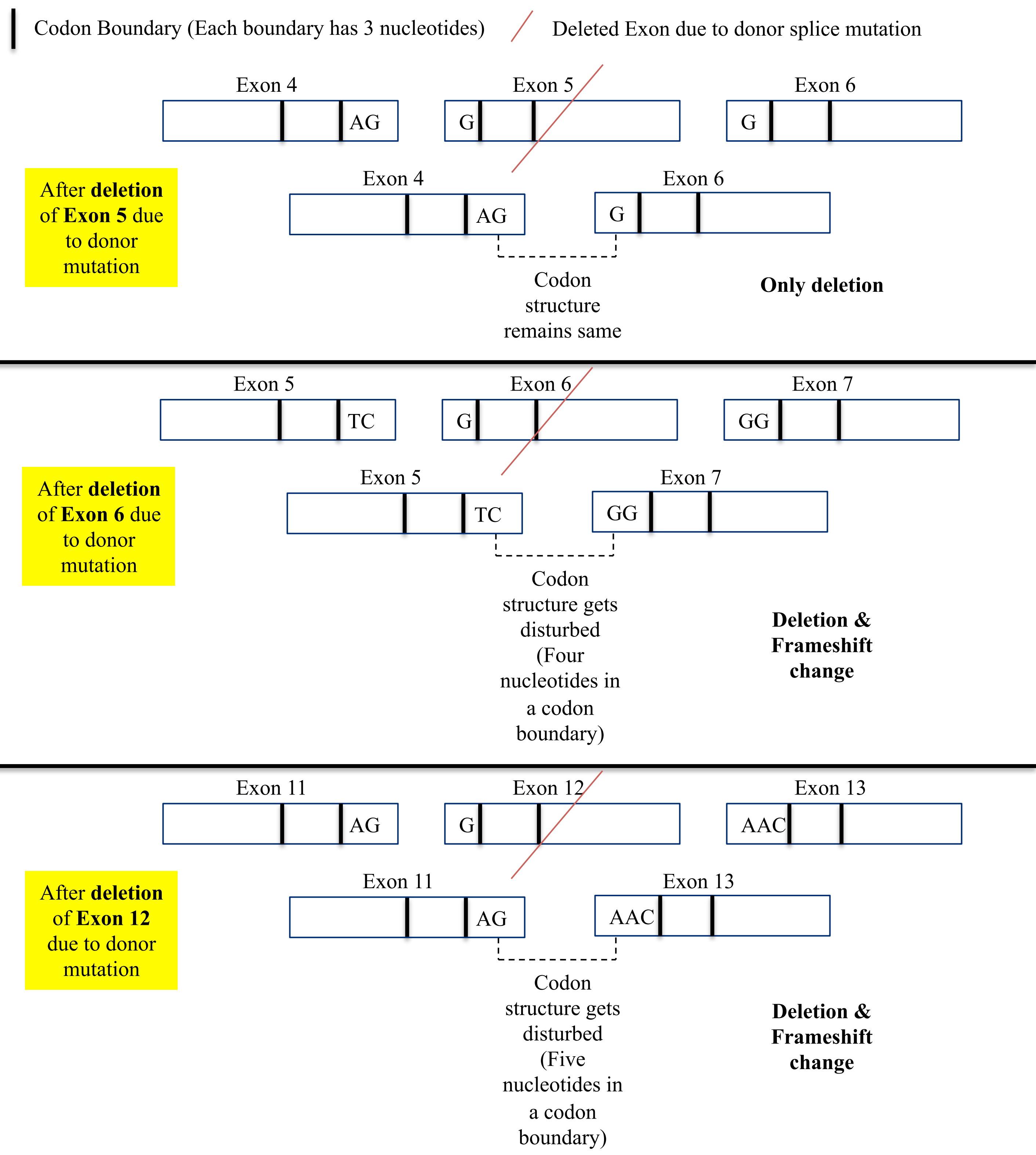

Codon Boundary (Each boundary has 3 nucleotides)
Deleted Exon due to donor splice mutation
Exon 4
AG
Exon 5
G
Exon 6
G
After deletion of Exon 5 due to donor mutation
Exon 4
AG
Exon 6
G
Codon structure remains same
Only deletion
Exon 5
TC
Exon 6
G
Exon 7
GG
After deletion of Exon 6 due to donor mutation
Exon 5
TC
Exon 7
GG
Codon structure gets disturbed (Four nucleotides in a codon boundary)
Deletion & Frameshift change
Exon 11
AG
Exon 12
G
Exon 13
AAC
After deletion of Exon 12 due to donor mutation
Exon 11
AG
Exon 13
AAC
Codon structure gets disturbed (Five nucleotides in a codon boundary)
Deletion & Frameshift change
